# The dose-response relationship of serum uric acid with Dyslipidaemia and its components: a cross-sectional study of a Chinese multi-ethnic cohort

**DOI:** 10.1186/s12944-022-01647-5

**Published:** 2022-04-03

**Authors:** Lian Peng, Leilei Liu, Nana Ma, Fan Yang, Chan Nie, Tingting Yang, Qibing Zeng, Ziyun Wang, Degan Xu, Lu Ma, Yuyan Xu, Feng Hong

**Affiliations:** 1 School of Public Health, The Key Laboratory of Environmental Pollution Monitoring and Disease Control, Ministry of Education, Guizhou Medical University, Dongqing Road, Guian New Area, Guiyang, 550025 People’s Republic of China; 2 Guiyang Center for Disease Control and Prevention, Guiyang, 550003 China

**Keywords:** Serum uric acid, Dyslipidaemia, Dose-response relationship, Risk factor

## Abstract

**Background:**

The association between serum uric acid (SUA) and the components of dyslipidaemia and their dose-response relationships have not been thoroughly explored. This study assessed the relationship between SUA and each dyslipidaemia component in Dong, Miao, and Bouyei populations in Guizhou by sex and ethnicities and investigated the dose-response relationship.

**Methods:**

In total, 16,092 participants aged 30–79 years from The China Multi-Ethnic Cohort (CMEC) Study were examined. Multivariable logistic regression models were applied to explore the relationship between SUA and each dyslipidaemia component by sex and three ethnicities. The dose-response associations between SUA and various dyslipidaemias were investigated using restricted cubic spline regression.

**Results:**

After controlling for confounding factors, the SUA level in total participants positively correlated with each dyslipidaemia component, and women had higher odds ratios (ORs) for each dyslipidaemia component than men (*P* for trend < 0.001). At the SUA level > 6.37 mg/dL, ORs (95% CI) for dyslipidaemia in the Dong, Miao and Bouyei were 2.89 (2.00–4.19), 2.43 (1.70–3.48), and 3.26 (2.23–4.78), respectively. When the SUA concentration increased by 1 mg/dL, the ORs (95% CI) for total dyslipidaemia was 1.31 (1.24–1.37). A positive dose-response but nonlinear association was found between SUA and total dyslipidaemia, high total cholesterol, and low HDL, whereas an inverse U-shaped association was found between SUA and high LDL-C ( *P*-nonlinear< 0.0001).

**Conclusion:**

The SUA level was positively correlated with each dyslipidaemia component in Dong, Miao, and Bouyei adults, and sex and ethnic differences were also found. A nonlinear dose-response relationship was found between SUA levels and dyslipidaemia and its components. Further research is warranted to investigate the causal link between SUA levels and dyslipidaemia incidence.

## Introduction

According to the global burden of disease study, metabolic risk variables such as dyslipidaemia are the most important determinants of noncommunicable diseases worldwide [1]. Dyslipidaemia is described as an aberrant lipid profile and characterized by high triglyceride (TG), total cholesterol (TC), low-density lipoprotein cholesterol (LDL-C), or high-density lipoprotein cholesterol (HDL-C) levels; globally, it is the leading cause of death [2]. The incidence of dyslipidaemia has been increasing in China in recent years [3, 4]. It has been reported that the age- and sex-standardized prevalence of dyslipidaemia in the Chinese population was as high as 43% [5]. Several prospective cohort studies have shown that HDL-C abnormalities increase the risk of death [6, 7]. Several studies have demonstrated that the effective dyslipidaemia treatment is a priority in cardiovascular prevention because early prevention and management of dyslipidaemia can help prevent cardiovascular events [8, 9]. Thus, identifying important risk factors, such as SUA levels, is critical for controlling the incidences of dyslipidaemia and related diseases [10, 11]. Due to the lack of uricase in humans, elevated SUA levels are the risk factors for multiterritorial atherosclerosis [12], diabetes [13], metabolic syndrome [14], and cardiovascular mortality [15].

The relationship between SUA and dyslipidaemia is complicated and is yet to be fully elucidated [11, 16]. SUA has been associated with dyslipidaemia in several studies [10, 16–19]. Some of the studies have confirmed that the association of SUA with components of dyslipidaemia is controversial [10, 20–22]. A study indicated a substantial positive relationship between SUA and HDL-C [23], whereas a cross-sectional study from China found no link between SUA levels and dyslipidaemia [24]. In terms of sex, some studies have shown no association between SUA and dyslipidaemia in women [25]; however, other studies have reported this association in both male and female populations [11]. Despite a cohort study that investigated the dose-response relationship of SUA with TC and TG [18], to the best of our knowledge, there is presently a paucity of information on dose-response relationship between SUA and dyslipidaemia components.

With 56 ethnic groups, China is a multi-ethnic country. Most studies on the relationship between SUA and each component of dyslipidaemia have focused on a single ethnic group [23]. Therefore, the present study aimed to assess the association of SUA levels with dyslipidaemia and each component of dyslipidaemia according to the total population, sex, and three ethnicities (Dong, Miao, and Bouyei in Guizhou Province, China) and investigate the possible dose-response relationship.

## Materials and methods

### Study population and design

**Fig. 1:**
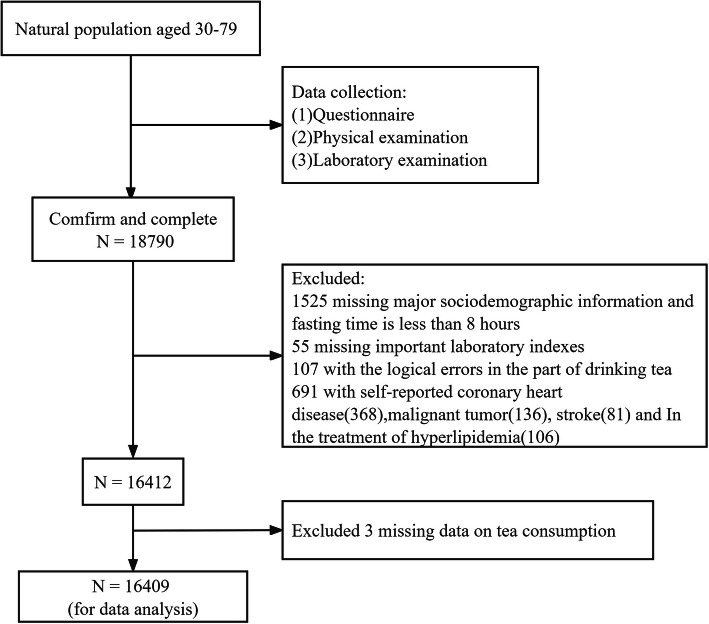
Flow chart of the research object

Between July 2018 and April 2019, participants were enrolled in a cross-sectional study based on the CMEC study in Guizhou Province, China, by using a multistage stratum cluster sampling method [26]. Inclusion criteria for selection of participants were: (i) individuals aged 30–79 years; (ii) permanent residents capable of completing baseline surveys and follow-up studies; (iii) individuals participating voluntarily in the study, providing consent to collect their biological samples, and having signed the informed consent. Exclusion criteria included: (i) serious physical or mental illness; (ii) failure to comply with research requirements. (iii) missing data for plasma and fasting blood glucose. A total of 18,790 individuals participated in the baseline survey, completing a touch screen questionnaire and providing physical measurements and biological samples, as detailed elsewhere [27]. Given that certain conditions or treatments may affect dyslipidaemia incidence or SUA levels, 1528 people whose major sociodemographic information was not available and who had a fasting time of less than 8 h were excluded. Additionally, 55 people whose important laboratory indices were lacking, and 107 individuals whose important variables exhibited logical errors were excluded. Moreover, this study excluded 691 individuals with self-reported coronary heart disease (*n* = 368), malignant tumor (*n* = 136), stroke (*n* = 81), and hyperlipidemia (*n* = 106), and 317 individuals with extreme UA values (by using the method to eliminate data < 1 and > 99%). Thus, the final analysis comprised 16,092 individuals (Fig. 1).

Every participant provided written informed consent before participating in the baseline survey. The Sichuan University Medical Ethical Review Board (K2016038) and the Research Ethics Committee of The Affiliated Hospital of Guizhou Medical University approved the study (2018[094]).

### Assessment of covariants

Data related to sociodemographic characteristics and health-related behaviors such as sex, ethnicity, age, area, education level, occupation (retiree/unemployed workers, professional skill workers, institutions, manual workers), smoking status, alcohol drinking status, tea drinking, physical activity, incidences of heart disease, stroke, and malignant tumor, and details on the treatment of hyperlipidaemia, were collected through face-to-face interviews. The investigation’s details have been revealed in previous studies [27]. A qualitative food frequency questionnaire was conducted to assess habitual dietary consumption (red meat, fresh fruits, and fresh vegetables) in the previous 12 months [28]. Alcohol drinking was considered as drinking frequency at least once a week for at least a year. Tea drinking was considered as the tea-drinking frequency at least once a week for more than 6 months. The metabolic equivalent task hours/days of activities linked to occupation, transportation, housekeeping, and leisure were added up to determine physical activity [29]. The total energy consumption per week in kilocalories (kcal/week) was defined as energy intake, and the specific calculation method detailed in previous research was used [30].

Physical examination was performed by healthcare professionals using standard methods. The average of three blood pressure (BP) readings collected using an electronic sphygmomanometer at 5-min intervals was recorded. The diagnostic criteria for hypertension were: systolic BP ≥ 140 mmHg; measured diastolic BP ≥ 90 mmHg; a previous diagnosis of hypertension; or use of anti-hypertensive medication [31]. Fasting blood glucose ≥7.0 mmol/L and random blood glucose ≥11.1 mmol/L were used to diagnose the diabetes epidemic [32]. Body mass index (BMI) was defined as weight divided by height (kg/m^2^).

### Lipid and SUA measurements

Professionally trained nurses collected blood samples under stringent aseptic conditions from the participants who fasted overnight (at least 8 h). The samples were clotted, and serum was separated on-site through centrifugation; the samples were immediately transported to the KingMed Diagnostics Group Co. Ltd., Guizhou Province, for biochemical analysis. Serum was isolated to analyze biochemical markers such as SUA, FBG, TC, TG, HDL-C, and LDL-C by using an automatic biochemical analyser (HITACHI 7180, Tokyo, Japan). This study adopted strict quality control procedures and performed standardized ability tests. This study used the cholesterol oxidase method to measure the TC value, the direct method to measure the HDL-C and LDL-C values, and the enzymatic method to measure the TG value.

### Diagnostic criteria

According to the 2016 Chinese Adult dyslipidaemia Prevention and Treatment Guidelines [28], TG ≥ 2.26 mmol/L(100 mg/dL) was considered high TG, TC ≥ 6.22 mmol/L(240 mg/dL) was considered as high TC, HDL-C < 1.04 mmol/L(40 mg/dL) was considered as low HDL-C, and LDL-C ≥ 4.14 mmol/L (160 mg/dL) was considered as high LDL-C. Dyslipidaemia was diagnosed in patients who had one of the aforementioned diseases or were diagnosed in a hospital at or above the district/county level and were currently using lipid-lowering medicines.

### Statistical analyses

All continuous variables in this study exhibited non-normal distributions; therefore, these variables are presented as median (interquartile range), whereas categorical variables are presented as frequency (percentage). The Kolmogorov-Smirnov test was used to verify normality of the data. The Mann-Whitney *U* test, Kruskal-Wallis *H* test, and Chi-square test were used to compare participant characteristics. First, this study conducted basic characteristic analyses for dyslipidaemia status and the quartile array of SUA (the lowest group was used as the reference group for analysis). To minimize information loss and investigate the true correlation between SUA and dyslipidaemia, this study considered SUA as a continuous variable and computed ORs and 95% confidence intervals (95%CIs) of SUA for each 1 mg/dL increase according to sex and ethnicities separately. The multivariable logistic regression model was used to examine the relationship of SUA quartiles with continuous variables and various dyslipidaemia components, with adjustments for ethnicity, sex, area, age(years), educational level, occupation, smoking status, alcohol drinking status, tea, hypertension, diabetes incidence, red meat intake, BMI, physical activity, energy, and waist and hip circumference. In addition, this study used the restricted cubic spline (RCS) regression method to visually investigate the dose-response relationships between SUA and different dyslipidaemia outcomes. IBM SPSS version 22.0(SPSS Inc.) and STATA version 12.0 were used for statistical analyses (Stata Corp). Based on the two-sided test level of *α* = 0.05, a *P* value of < 0.05 was considered to denote statistical significance.

## Results

### Analysis of basic characteristics of the study population

Table 1 shows the baseline demographic and clinical features of the research population according to dyslipidaemia status. The mean age of individuals who did not develop dyslipidaemia was 50.8(43.5–60.3), whereas the mean age of those who developed dyslipidaemia was 52.5(45.7–60.0)(*P* < 0.001). The difference in dyslipidaemia prevalence across sex and ethnic groups was statistically significant (*P* < 0.001). Participants with dyslipidaemia were more likely to be female and Dong and rural adults, with a poor educational level; a high occupation level; diabetes; high SUA level, BMI, energy, and waist and hip circumference; and low physical activity levels.

**Table 1:** Baseline characteristics according to their dyslipidaemia status

	Non-dyslipidaemia	Dyslipidaemia	***P*** value
	11,109 (69.0%)	4983 (31.0%)	
Ethnicity, n (%)			< 0.001
Dong	4157 (37.4%)	2096 (42.1%)	
Bouyei	3768 (33.9%)	1321 (26.5%)	
Miao	3184 (28.7%)	1566 (31.4%)	
Sex, n (%)			< 0.001
Male	3281 (29.5%)	2121 (42.6%)	
Female	7828 (70.5%)	2862 (57.4%)	
Area			< 0.001
Rural	8978 (80.9%)	3783 (76.1%)	
Urban	2114 (19.1%)	1185 (23.9%)	
Age (years)	50.8 (43.5–60.3)	52.5 (45.7–60.0)	< 0.001
Educational level, n (%)			< 0.001
Low	6882 (61.9%)	2905 (58.3%)	
Medium	3226 (29.0%)	1532 (30.7%)	
High	1001 (9.0%)	546 (11.0%)	
Occupation, n (%)			< 0.001
low	3219 (29.0%)	1576 (31.7%)	
Medium	1520 (13.7%)	775 (15.6%)	
High	6357 (57.3%)	2621 (52.7%)	
Smoking status, n (%)			< 0.001
Never	9153 (82.4%)	3742 (75.1%)	
Past	364 (3.3%)	207 (4.2%)	
Current	1592 (14.3%)	1034 (20.8%)	
Alcohol drinking status, n (%)			0.003
Yes	351 (3.2%)	203 (4.1%)	
No	10,758 (96.8%)	4780 (95.9%)	
Tea, n (%)			< 0.001
Yes	1272 (11.5%)	729 (14.6%)	
No	9837 (88.5%)	4254 (85.4%)	
High blood pressure, n (%)			< 0.001
Yes	2618 (23.7%)	1821 (36.7%)	
No	8449 (76.3%)	3142 (63.3%)	
Diabetes, n (%)			< 0.001
Yes	703 (6.3%)	789 (15.9%)	
No	10,371 (93.7%)	4181 (84.1%)	
Red meat, g/week	700 (350–1050)	700 (350–1400)	0.013
BMI, kg/m^2^	23.4 (21.2–25.8)	25.4 (23.3–27.5)	< 0.001
Physical activity, met/day	25.0 (14.0–38.3)	22.8 (12.2–36.0)	< 0.001
Total energy intake, kcal/week	10,311.8 (8059.3–13,270.1)	10,701.2 (8335.1–13,747.2)	< 0.001
Waist circumference, cm	80.6 (74.0–87.4)	87.0 (81.1–93.0)	< 0.001
Hips, cm	91.0 (87.0–95.0)	93.5 (89.5–97.0)	< 0.001
Fresh fruits, g/week	2100.0 (1400.0–2800.0)	2100.0 (1400.0–2800.0)	0.149
Fresh vegetables, g/week	400.0 (105.0–900.0)	400.0 (100.0–900.0)	0.356
SUA (mg/dL)	5.0 (4.2–6.1)	5.9 (4.9–7.0)	< 0.001
TC, mmol/L	4.7 (4.2–5.2)	5.6 (4.7–6.3)	< 0.001
TG, mmol/L	1.2 (0.9–1.6)	2.6 (2.0–3.5)	< 0.001
HDL-C, mmol/L	1.5 (1.3–1.7)	1.3 (1.1–1.6)	< 0.001
LDL-C, mmol/L	2.6 (2.2–3.1)	3.1 (2.4–4.0)	< 0.001
Fasting blood glucose, mmol/L	5.2 (4.9–5.9)	5.4 (5.0–5.9)	< 0.001
Glycated haemoglobin, %	5.2 (5.2–5.0)	5.7 (5.4–6.1)	< 0.001

Continuous variables are shown as medians (interquartile range), and categorical variables are shown as frequencies (percentages). Comparison of participant characteristics in each group was performed using the Mann-Whitney *U* test or Chi-square test

### Baseline characteristics of participants by SUA quartiles and sex

The characteristics of the participants were stratified by quartile of SUA levels and sex (Table 2). The SUA quartiles were: Q1 ≤ 4.39 mg/dL; 4.39 mg/dL < Q2 ≤ 5.29 mg/dL; 5.29 mg/dL < Q3 ≤ 6.36 mg/dL; 6.37 mg/dL < Q4. Ethnicity, area, age, educational level, occupation, high BP, diabetes, BMI, physical activity, waist circumference, and hip circumference were associated with the SUA quartile (all *P* < 0.05). The SUA quartiles of different sexes had statistical differences in ethnicity, area, age, educational level, occupation, high BP, diabetes, BMI, physical activity, waist circumference, and hip circumference (all *P* < 0.05). With the increase in SUA levels, BMI, drinking frequency, tea-drinking frequency, percentage of individuals with high BP, waist circumference, hip circumference, and fresh fruit intake also increased, whereas the percentage of Bouyei adults and physical activity declined. For men, smoking and alcohol drinking frequencies did not vary statistically among between different quartile groups. For women, with the increase in SUA levels, age and the percentage of urban residents, Dong adults, individuals with high educational levels, and those with low occupation also increased, whereas rural residence and total energy intake declined. The higher the quartile of SUA, the higher were the proportions of dyslipidaemia, hypertension, and diabetes (*P* < 0.001, respectively).

**Table 2:** Baseline characteristic of the participants by SUA quartile categories and sex

Quartile of SUA-Men						Quartile of SUA-Women				
	Q1 (***n*** = 1351) (≤5.45 mg/dL)	Q2 (***n*** = 1347) (5.46–6.38 mg/dL)	Q3 (***n*** = 1341) (6.39–7.40 mg/dL)	Q4 (***n*** = 1363) (≥7.41 mg/dL)	***P*** value	Q1 (***n*** = 3982) (≤ 4.14 mg/dL)	Q2 (***n*** = 4098) (4.15–4.83 mg/dL)	Q3 (***n*** = 4099) (4.84–5.69 mg/dL)	Q4 (***n*** = 3913) (≥5.70 mg/dL)	***P*** value
Ethnicity, n (%)					< 0.001					< 0.001
Dong	489 (36.2%)	493 (36.6%)	586 (43.7%)	591 (43.4%)		957 (35.3%)	989 (37.6%)	1012 (37.9%)	1136 (42.4%)	
Bouyei	455 (33.7%)	393 (29.2%)	338 (25.2%)	340 (24.9%)		1074 (39.6%)	903 (34.4%)	847 (31.7%)	739 (27.6%)	
Miao	407 (30.1%)	461 (34.2%)	417 (31.1%)	432 (31.7%)		683 (25.2%)	735 (28.0%)	811 (30.4%)	804 (30.0%)	
Area					< 0.001					< 0.001
Rural	1130 (83.8%)	1047 (78.0%)	984 (73.7%)	1013 (74.5%)		2316 (85.4%)	2156 (82.2%)	2083 (78.1%)	2032 (76.0%)	
City	219 (16.2%)	296 (22.0%)	351 (26.3%)	347 (25.5%)		395 (14.6%)	468 (17.8%)	583 (21.9%)	640 (24.0%)	
Age (years)	54.0 (46.6–63.9)	52.5 (44.7–62.3)	52.0 (44.2–61.9)	52.7 (44.5–62.3)	< 0.001	49.0 (42.3–56.1)	49.6 (42.9–56.2)	51.0 (44.0–58.5)	53.8 (46.3–62.2)	< 0.001
Educational level, n (%)					< 0.001					< 0.001
Low	747 (55.3%)	651 (48.3%)	592 (44.1%)	633 (46.4%)		1908 (70.3%)	1753 (66.7%)	1693 (63.4%)	1810 (67.6%)	
Medium	475 (35.2%)	512 (38.0%)	534 (39.8%)	504 (37.0%)		629 (23.2%)	676 (25.7%)	755 (28.3%)	673 (25.1%)	
High	129 (9.5%)	184 (13.7%)	215 (16.0%)	226 (16.6%)		177 (6.5%)	198 (7.5%)	222 (8.3%)	196 (7.3%)	
Occupation, n (%)					< 0.001					< 0.001
low	286 (21.2%)	281 (20.9%)	324 (24.2%)	327 (24.1%)		725 (26.8%)	782 (29.8%)	931 (34.9%)	1139 (42.5%)	
medium	165 (12.2%)	230 (17.1%)	262 (19.6%)	262 (19.3%)		334 (12.3%)	368 (14.0%)	372 (14.0%)	302 (11.3%)	
High	897 (66.5%)	832 (62.0%)	753 (56.2%)	770 (56.7%)		1650 (60.9%)	1476 (56.2%)	1362 (51.1%)	1238 (46.2%)	
Smoking status, n (%)					0.497					0.780
Never	569 (42.1%)	565 (41.9%)	573 (42.7%)	553 (40.6%)		2703 (99.6%)	2611 (99.4%)	2652 (99.3%)	2669 (99.6%)	
Past	131 (9.7%)	129 (9.6%)	153 (11.4%)	149 (10.9%)		3 (0.1%)	0 (0.0%)	2 (0.1%)	4 (0.1%)	
Current	651 (48.2%)	653 (48.5%)	615 (45.9%)	661 (48.5%)		8 (0.3%)	16 (0.60%)	16 (0.60%)	6 (0.2%)	
Alcohol drinking status, n (%)					0.331					0.005
Yes	80 (5.9%)	92 (6.8%)	94 (7.0%)	105 (7.7%)		30 (1.1%)	39 (1.5%)	53 (2.0%)	61 (2.3%)	
No	1271 (94.1%)	1255 (93.2%)	1247 (93.0%)	1258 (92.3%)		2684 (98.9%)	2588 (98.5%)	2617 (98.0%)	2618 (97.7%)	
Tea, n (%)					< 0.001					0.306
Yes	229 (17.0%)	293 (21.8%)	296 (22.1%)	366 (26.9%)		190 (7.0%)	193 (7.3%)	215 (8.1%)	219 (8.2%)	
No	1122 (83.0%)	1054 (78.2%)	1045 (77.9%)	997 (73.1%)		2524 (93.0%)	2434 (92.7%)	2455 (91.9%)	2460 (91.8%)	
High blood pressure, n (%)					< 0.001					< 0.001
Yes	402 (29.9%)	433 (32.2%)	492 (36.8%)	638 (46.9%)		483 (17.9%)	509 (19.4%)	612 (23.1%)	870 (32.6%)	
No	941 (70.1%)	911 (67.8%)	846 (63.2%)	721 (53.1%)		2219 (82.1%)	2113 (80.6%)	2043 (76.9%)	1797 (67.4%)	
dyslipidaemia, n (%)					< 0.001					< 0.001
Yes	343 (25.4%)	458 (34.0%)	581 (43.3%)	739 (54.2%)		433 (16.0%)	580 (22.1%)	759 (28.4%)	1090 (40.7%)	
No	1008 (74.6%)	889 (66.0%)	760 (56.7%)	624 (45.8%)		2281 (84.0%)	2047 (77.9%)	1911 (71.6%)	1589 (59.3%)	
Diabetes, n (%)					< 0.001					< 0.001
Yes	212 (15.8%)	146 (10.9%)	137 (10.2%)	186 (13.7%)		150 (5.5%)	144 (5.5%)	177 (6.6%)	340 (12.7%)	
No	1131 (84.2%)	1190 (89.1%)	1201 (89.8%)	1173 (86.3%)		2561 (94.5%)	2479 (94.5%)	2485 (93.4%)	2332 (87.3%)	
Red meat, g/week	700.0 (350.0–1400.0)	700.0 (400.0–1400.0)	700.0 (420.0–1400.0)	700.0 (420.0–1400.0)	0.432	700.0 (350.0–1050.0)	700.0 (350.0–1050.0)	700.0 (350.0–1050.0)	700.0 (350.0–1050.0)	0.481
BMI, kg/m^2^	22.6 (20.7–25.0)	23.7 (21.5–25.9)	24.6 (22.3–26.7)	25.6 (23.1–27.8)	< 0.001	23.5 (21.4–25.8)	23.5 (21.4–25.8)	24.4 (22.2–26.7)	25.7 (23.4–28.0)	< 0.001
Physical activity, met/day	26.7 (15.0–40.6)	25.6 (13.4–39.2)	23.6 (13.3–37.9)	22.3 (12.1–36.6)	< 0.001	26.6 (15.7–39.1)	25.3 (15.2–38.2)	24.1 (12.9–36.8)	21.1 (10.7–33.6)	< 0.001
Total energy intake, kcal/week	11,751.4 (8930.7–14,843.1)	11,626.4 (9071.7–15,009.2)	11,639.8 (9274.3–14,619.6)	11,926.9 (9362.9–14,864.8)	0.546	10,033.5 (7750.8–12,613.7)	10,023.1 (7901.5–12,766.0)	9851.2 (7511.4–12,554.9)	9798.0 (7701.5–12,309.9)	0.490
Waist circumference, cm	80.0 (74.0–87.0)	83.2 (76.5–89.4)	86.0 (79.2–92.0)	89.0 (82.5–95.0)	< 0.001	78.0 (72.0–84.0)	80.5 (73.7–86.8)	83.0 (76.5–89.0)	86.5 (80.1–93.0)	< 0.001
Hips circumference, cm	90.5 (86.5–94.0)	92.0 (88.0–95.6)	93.0 (89.0–97.0)	94.0 (90.0–98.2)	< 0.001	90.0 (86.0–93.6)	91.0 (86.5–95.0)	92.0 (88.0–96.0)	93.0 (89.0–97.5)	< 0.001
Fresh fruits, g/week	240.0 (58.3–700.0)	300 (93.3–700.0)	300.0 (87.5–700.0)	350 (93.3–750.0)	< 0.001	400.0 (116.7–875.0)	450.0 (140.0–1050.0)	500.0 (140.0–11,260.0)	500.0 (140.0–1050.0)	< 0.001
Fresh vegetables, g/week	2100.0 (1400.0–3150.0)	2100.0 (1400.0–3150.0)	2100.0 (1400.0–2800.0)	2100.0 (1400.0–3150.0)	0.887	2100.0 (1400.0–2800.0)	2100.0 (1400.0–2800.0)	2100.0 (1400.0–3150.0)	2100.0 (1400.0–2800.0)	0.410

Continuous variables are shown as medians (interquartile range), and categorical variables are shown as frequencies (percentages). Participant characteristics in each group were compared using the Kruskal-Wallis *H* test or the Chi-square test

### Association between SUA levels and each component of dyslipidaemia in different subgroups

**Fig. 2:**
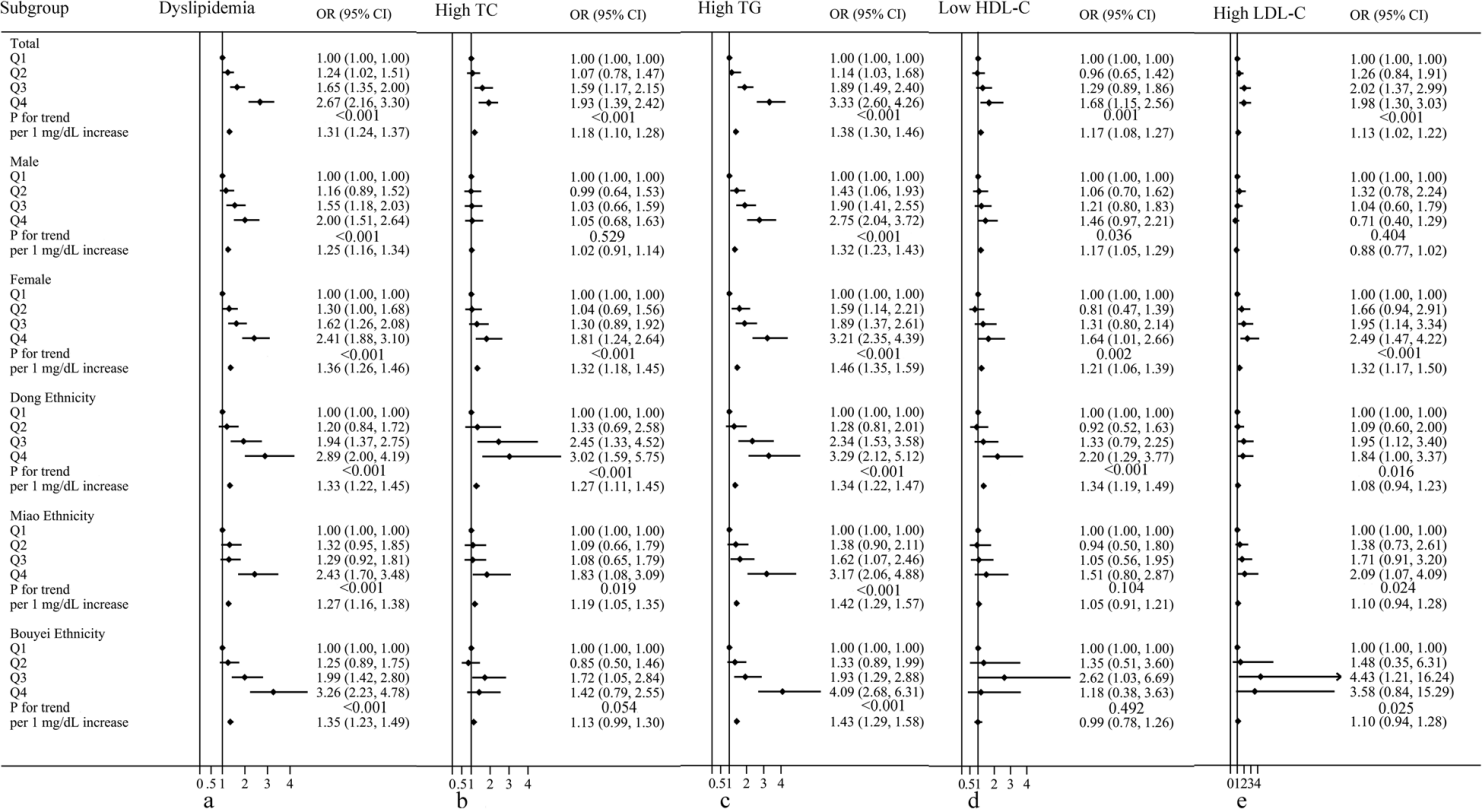
Multivariate analysis of the relationship between serum uric acid and dyslipidaemia and each component of dyslipidaemia, after adjusting for variable (except for the stratified variables) such as ethnicity, gender, area, age, educational level, occupation, smoking status, alcohol drinking status, tea, hypertension, diabetes, red meat, body mass index, physical activity, total energy intake, waist circumference, and hip circumference

Figure 2 shows that in total participants, the SUA level was substantially, positively, and linearly associated with overall dyslipidaemia, high TC, high TG, and low HDL-C (*P* for trend < 0.001). Compared with the ORs of the first quartile of SUA levels in the SUA quartile category of all participants, the ORs of the second, third, and fourth quartiles for overall dyslipidaemia were 1.24(1.02–1.51), 1.65(1.35–2.00), and 2.67(2.16–3.30), respectively; those for high TC were 1.07(0.78–1.47), 1.59(1.17–2.15), and 1.93(1.39–2.42), respectively; those for high TG were 1.14(1.03–1.68), 1.89(1.49–2.40), and 3.30(2.60–4.26), respectively; those for low HDL-C were 0.96(0.65–1.42), 1.29(0.89–1.86), and 1.68(1.15–2.56), respectively; and those for high LDL-C were 1.26(0.84–1.19), 2.02(1.37–2.99), and 1.98(1.30–3.03), respectively. According to the findings, people with greater SUA levels were more likely to have overall dyslipidaemia, high TC, high TG, and low HDL-C. However, the probability of high LDL-C increased with an increase in the SUA level and subsequently reduced to a considerable amount (*P* for trend < 0.001).

In terms of sex, the ORs for each component of dyslipidaemia in women were greater than those in men, with an increase in the SUA quartile, indicating that women were at a higher risk of having dyslipidaemia than men. Except for high TC and high LDL-C in men, SUA levels and total dyslipidaemia, high TG, and low HDL-C continued to demonstrate a linear trend in the sex subgroup (*P* for trend < 0.05). Surprisingly, the pattern of progressively increasing and then decreasing LDL-C levels with SUA levels was observed in both total participants and men and Dong and Bouyei participants. When the SUA level was > 6.37 mg/dL, the Dong adults had a higher risk of total dyslipidaemia and high TC than the Miao and Bouyei adults; the ORs (95%CI) for total dyslipidaemia in the Dong, Miao and Bouyei were 2.89(2.0–4.19), 2.43(1.70–3.48), and 3.26(2.23–4.78), respectively; the ORs (95%CI) for high TC in the Dong, Miao and Bouyei were 3.02(1.59–5.75), 1.83(1.08–3.09), and 1.42(0.79–2.55), respectively. Nevertheless, the Bouyei adults were more likely to have high LDL-C than the Dong and Miao adults; the ORs (95%CI) for high LDL-C in the Dong, Miao and Bouyei were 1.84(1.00–3.37), 2.09(1.07–4.09), and 3.58(0.84–15.29), respectively.

The risk of different dyslipidaemias was strongly associated with the SUA level (the continuous variable). This relationship remained substantial after controlling for sex and ethnic subgroups. When SUA increased by 1 mg/dL, the OR (95%CI) for overall dyslipidaemia was 1.31(1.24–1.37) (*P* < 0.001), with the OR of 1.25(1.16–1.34) (*P* < 0.001) for men and that of 1.36(1.26–1.46) (*P* < 0.001) for women. The OR (95%CI) for high TC associated with a 1 mg/dL increase in SUA concentration was 1.18(1.10–1.28) (*P* < 0.001), with the OR of 1.02(0.91–1.14) (*P* = 0.703) for men and that of 1.32(1.18–1.45) (*P* < 0.001) for women. When SUA increased by 1 mg/dL, the OR (95%*CI*) for high TG was 1.38(1.30–1.46) (*P* < 0.001), with the OR of 1.32(1.23–1.43) (*P* = 0.703) for men and that of 1.46(1.35–1.59) (*P* < 0.001) for women. The OR (95%CI) for low HDL-C associated with a 1 mg/dL increase in SUA concentration was 1.17(1.08–1.27) (*P* < 0.001), with the OR of 1.17(1.05–1.29) (*P* = 0.003) for men and that of 1.21(1.06–1.39) (*P* = 0.006) for women. When SUA increased by 1 mg/dL, the OR (95%*CI*) for high LDL-C was 1.13(1.02–1.22) (*P* = 0.023), with the OR of 0.88(0.77–1.02) (*P* = 0.079) for men and that of 1.32(1.17–1.50) (*P* < 0.001) for women.

### The dose-response association between SUA and dyslipidaemia

**Fig. 3:**
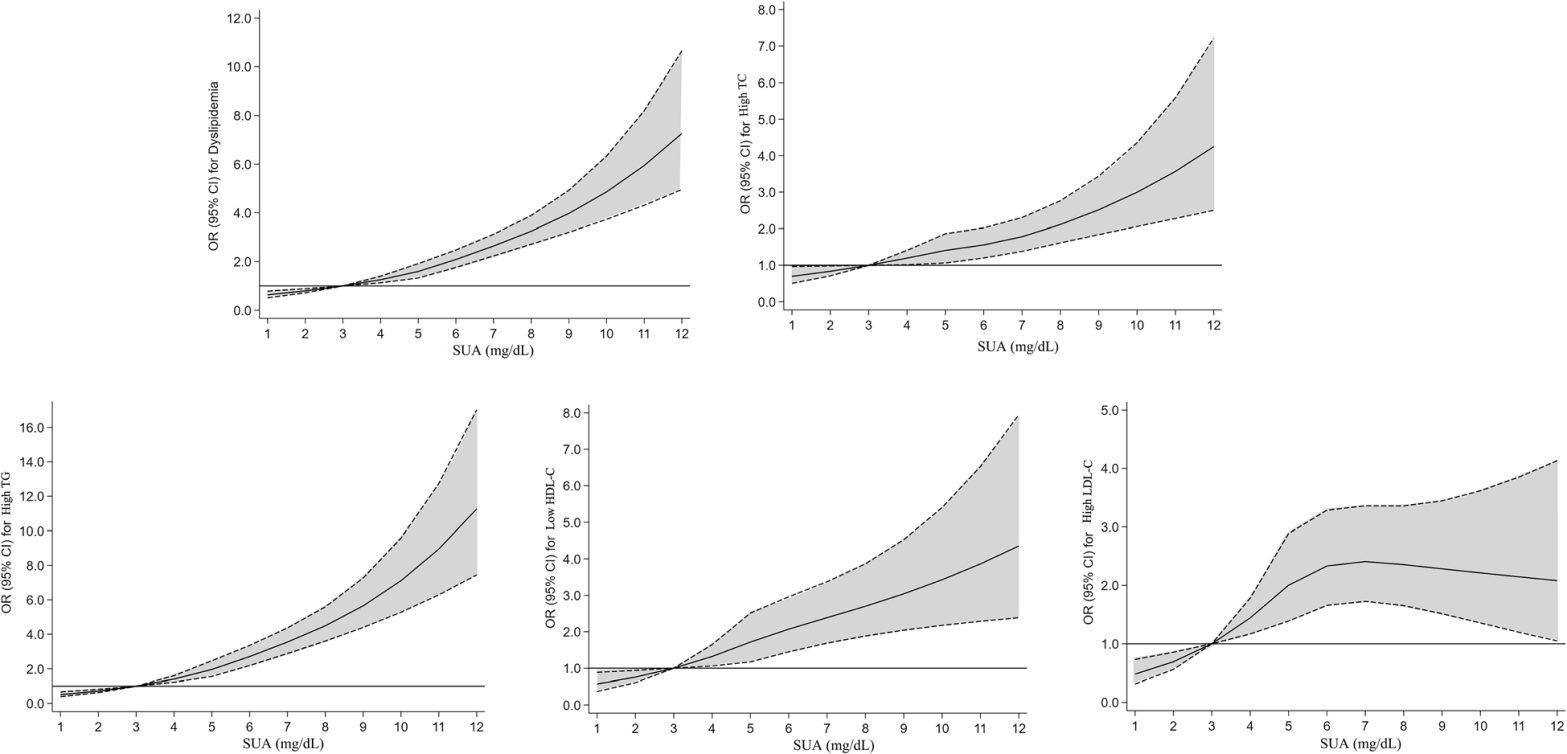
The dose-response relationship between serum uric acid and dyslipidaemia and each component of dyslipidaemia, after adjusting for variables (except for the stratified variables) such as ethnicity, gender, area, age, educational level, occupation, smoking status, alcohol drinking status, tea, hypertension, diabetes, red meat, body mass index, physical activity, total energy intake, waist circumference, and hip circumference

The dose-response relationship between SUA and the risk of each dyslipidaemia component was examined in the total participants by using RCS regression with four knots after multivariable regression analysis. The RCS analysis revealed a positive dose-response but nonlinear association between the SUA level and total dyslipidaemia (*P*-nonlinear< 0.0001), high TC (*P*-nonlinear< 0.0001), high TG (*P*-nonlinear< 0.0001), and low HDL-C (*P*-nonlinear< 0.0001) in the total participants (Fig. 3). The risk of dyslipidaemia increased in a nonlinear fashion with an increase in the SUA level after controlling for confounders. The results of high LDL-C showed a completely different trend, with the RCS demonstrating an inverse U-shaped relationship between SUA levels and high LDL-C risk (*P*-nonlinear< 0.0001); the ORs for high LDL-C increased sharply and then decreased gradually when the SUA level was approximately > 6 mg/dL.

## Discussion

This study mainly shows that the SUA level is related strongly to total dyslipidaemia, high TC, high TG, low HDL-C, and high LDL-C, despite sex and ethnicity variations. Furthermore, the current study discovered that in the SUA quartile group, the ORs for dyslipidaemia and each component of dyslipidaemia increased in total participants and displayed linear trends. After controlling for potential confounding factors, a nonlinear upward trend in the dose-response relationship was found between SUA and total dyslipidaemia, high TC, high TG, and low HDL-C, whereas an inverse U-shaped dose-response relationship was found between SUA levels and high LDL-C. These results add to the existing epidemiological evidence for the relationship between SUA and various dyslipidaemia components. Additionally, these findings may have important public health implications.

### Comparisons with other studies

Previous research has indicated a substantial relationship between SUA and dyslipidaemia [10, 11, 19]. However, the reported specific positive or negative associations between SUA and the various components of dyslipidaemia (total dyslipidaemia, high TC, high TG, low HDL-C, and high LDL-C) are debatable and warrant further investigation. Some studies, for example, have found that SUA is positively associated with low HDL-C [25, 33], whereas other studies have found no such association [24, 34]. SUA was positively correlated with total dyslipidaemia, high TC, high TG, and low HDL-C in the present study. The risk of different dyslipidaemias was strongly associated with the SUA level (a continuous variable). Potential mechanisms may explain the harmful causal effects of SUA on dyslipidaemia: SUA was formerly thought to be an antioxidant; however, at high doses, it functions as a pro-oxidant molecule [25]. A high SUA level has been hypothesized to be associated with increased lipid peroxidation rates [35]. Despite mounting evidence from fundamental research, the specific mechanism through which SUA levels promote dyslipidaemia remains unknown. Notably, lipoprotein(a) [Lp(a)] is an important cardiovascular risk factor, and increasing evidence is available for a causal relationship between Lp(a) and cardiovascular events due to its pro-atherosclerotic LDL-C-like properties and prothrombotic plasminogen-like activity; a growing body of evidence also suggests a causal relationship between cardiovascular events and apolipoprotein(a) [36–38]. However, Lp(a) levels were not assessed in this study, and the association between Lp(a) and SUA deserves investigation in future studies.

In this study, the men had significantly higher SUA levels than the women in both dyslipidaemia and non-dyslipidaemia groups. This finding is consistent with that of a study [20]. This may be due to the role of steroids in SUA regulation, also known as the ʻuricosuric effectʼ, and the possible SUA inhibitory effect of oestrogen on women [39]. Although men have higher SUA levels than women, in the sex-stratified analysis, the association between SUA levels and various dyslipidaemia components appeared to be stronger in women. This result is completely different from those of previous studies that have reported no association between SUA and dyslipidaemia in women [25]. A cohort study found that the relationship between SUA and elevated TG levels was more pronounced in women [24]. Several investigations have found that the elevated SUA level is sex-dependent with cardiovascular factors [40]. Furthermore, a prospective study revealed that the association between hyperuricaemia and cardiovascular disease (CVD) mortality is greater in women than in men [41]. With every 1 mg/dL increase in the SUA level, the risk of dyslipidaemia is 1.31 times that of no dyslipidaemia. In women, SUA levels of > 5.70 mg/dL were associated with the highest risk of dyslipidaemia. Thus, this study urges that woman with SUA levels of > 5.70 mg/dL should be thoroughly evaluated to rule out the possibility of dyslipidaemia.

Among ethnic subgroups, Bouyei adults were more likely than Dong and Miao adults to have total dyslipidaemia and high LDL-C, whereas Dong adults were more likely than Miao and Bouyei adults to have high TC with increased SUA levels. Different lifestyle preferences may influence TC, HDL-C, LDL-C, and TG in individuals with dyslipidaemia [35, 42]. Different ethnic genes, social contexts, and dietary habits may also contribute to this disparity [43, 44]. Dong, Miao, and Bouyei individuals in Guizhou Province exhibit a distinct lifestyle. However, due to the design of the study, the effects of environment and genes on SUA and dyslipidaemia, as well as their correlations could not be investigated in this study. More studies are needed in the future to explore the influence of the living environment and genes in this specific domain.

In the present study, the distribution of dyslipidaemia in rural participants was higher than that in urban participants, which is different from the urban-rural dyslipidaemia distribution reported in a study [5]; this may be because most of the participants (79.3%) in this study were from rural areas. This research also indicated that people with diabetes, high BP, and tendency to smoke and drink alcohol and tea frequently accounted for a higher proportion of people with dyslipidaemia than of those without dyslipidaemia, and the finding is consistent with those of previous studies [45–47].

RCSs combine quantitative data with the correlation strength of the outcome to realize the continuous presentation of the correlation strength and dose-response relationship [48]. A recent meta-analysis found a favorable dose-response association between the SUA level and the risk of death from CVD [49]. After adjusting for possible confounders, a cohort study reported a positive linear dose-response association between SUA and high TG incidence [18]. However, this study found that the risk of high TG in the total population increased in a nonlinear fashion when SUA increased. Furthermore, the present study findings revealed a nonlinear upward dose-response relationship between SUA and overall dyslipidaemia, high TC, and low HDL in the total population. A cohort study in Japan found that the increased SUA level over time is a significant risk factor for high LDL-C and demonstrated a positive association between these variables [10]. The present RCS data revealed an inverse U-shaped correlation between SUA and the risk of high LDL-C; the ORs related to SUA and high LDL-C exhibited an increasing trend, reaching a peak when the SUA level exceeded approximately 6 mg/dL and subsequently showing a decreasing trend. A cohort study found a nonlinear and U-shaped association between SUA and mortality [50]. Another cohort study in the elderly population in China also showed a U-shaped relationship of the SUA levels with all-cause mortality and CVD mortality [51]. Although studies have shown an association between SUA levels and dyslipidaemia and its components [11, 19], the present study further showed a possible dose-response relationship, particularly in TC, LDL-C and HDL-C. The current findings reflect the importance of epidemiological data in determining the relationship between SUA and other components of dyslipidaemia.

### Clinical and public health potential

According to the existing literature, people at a risk of dyslipidaemia should actively manage their SUA levels. This study found a dose-response relationship between SUA and different dyslipidaemia components. Probably, the focus should be directed neither only on correcting dyslipidaemia while neglecting SUA nor solely on SUA that increases the risk of dyslipidaemia. Because SUA levels exhibit a substantial dose-response relationship with dyslipidaemia, establishing customized treatment guidelines for different sexes is critical to improve dyslipidaemia and overall long-term health consequences. The Dong, Miao and Bouyei ethnic groups in Guizhou Province have distinct eating habits and lifestyles. Specific health guidance can be formulated for areas with individuals of the three ethnic groups having a high SUA level; for example, studies have shown that animal viscera consumed by the Bouyei ethnic group and rice wine consumed by the Miao ethnic group increase the risk of hyperuricemia, whereas coarse grains and red acid soup protect against hyperuricemia [52]. This strategy can guide local residents to toward a healthy diet. A health and nutrition survey in China also showed that strengthening the control of the intake of animal-derived and soy foods is beneficial to control the risk of hyperuricemia [53]. In addition, researchers are placing increasing emphasis on the Mediterranean diet and lifestyle that involves high consumption of fruits and vegetables, limited meat intake, consumption of extra virgin olive oil and red wine, and regular physical activity [54]. Furthermore, both regular and high-intensity aerobic exercise appeared to be beneficial in improving the blood lipid status [55]. A randomized controlled study showed that moderate-intensity exercise (jogging) was more beneficial than low-intensity exercise (brisk walking) in reducing the risk of hyperuricemia [56]. Strategies capable of truly increasing the SUA levels and dyslipidaemia must be researched further. Furthermore, once an increase in SUA levels is noted, measures must be taken to avoid further increase to reduce the risk of dyslipidaemia. Clinically, special attention should be paid to the early detection and treatment of people with high SUA levels to prevent the incidence of dyslipidaemia.

### Strengths and limitations of the study

This study offers numerous advantages. Firstly, CMEC is the first large-scale cohort study that focused on ethnic minorities in China, and it used standardized laboratory tests, comprehensive body measurements, and professional questionnaires, which provide relatively strong evidence for assessing the specific association between SUA and different dyslipidaemia components. Second, this study explored the dose-response relationship between SUA and each dyslipidaemia component. These investigations address the limitations of earlier studies. Additionally, the present study conducted a specific analysis of SUA and the risk of each type of dyslipidaemia after controlling for various potential confounding factors, and the study also involved a subgroup analysis by sex and ethnicity. Furthermore, this study considered the SUA level as a continuous variable for analysis, which reduced information loss, and further explored the true association between SUA level and different dyslipidaemia components.

Despite these advantages, the present study has some shortcomings. For starters, the participants were from the Dong, Miao, and Bouyei ethnic groups in China; care was exercised when extrapolating the observed association to other ethnic groups because each ethnic group has different lifestyle. In addition, reverse causality is possible in every observational research [43]. Hence, the possibility of reverse causality between SUA levels and the type of dyslipidaemia cannot be ruled out. Finally, this study was based only on baseline data; therefore, the cumulative changes over time in the association between SUA and different dyslipidaemias could not be analyzed, and further data are needed to verify in the future.

## Conclusions

In conclusion, a positive correlation was observed between SUA levels and each component of dyslipidaemia (total dyslipidaemia, high TC, high TG, low HDL and high LDL-C) in Dong, Miao, and Bouyei adults, particularly in the women and the Bouyei adults. Sex and ethnic disparities should be considered while managing the blood lipid level. A nonlinear upward trend in dose-response correlation was found between SUA levels and total dyslipidaemia, high TC, and low HDL, whereas an inverse U-shaped association was found between SUA levels and the risk of high LDL-C. Special attention should be paid to the early detection and early treatment of people with high SUA levels to prevent the occurrence of dyslipidaemia. Appropriate treatment guidelines should be formulated through control measures such as those for diet, behavior, lifestyle, and drugs to prevent the increase in SUA levels, which can reduce the adverse effect of SUA on dyslipidaemia incidence. Further research is warranted to investigate the causal link between SUA levels and dyslipidaemia incidence.

## Data Availability

The data and materials of this study are available from the corresponding author upon reasonable request.

## Abbreviations

SUA: Serum uric acid
ORs: Odds ratios
TG: High triglyceride
TC: Total cholesterol
LDL-C: Low-density lipoprotein cholesterol
HDL-C: High-density lipoprotein cholesterol
CMEC: The China Multi-Ethnic Cohort
BP: Blood pressure
95% CIs: 95% confidence intervals
RCS: Restricted cubic spline
Lp(a): Lipoprotein(a)
CVD: Cardiovascular disease

## References

[1] Lim SS, Vos T, Flaxman AD, Danaei G, Shibuya K, Adair-Rohani H, Amann M, Anderson HR, Andrews KG, Aryee M (2012). A comparative risk assessment of burden of disease and injury attributable to 67 risk factors and risk factor clusters in 21 regions, 1990-2010: a systematic analysis for the global burden of disease study 2010. Lancet.

[2] Prabhakaran D, Anand S, Watkins D, Gaziano T, Wu Y, Mbanya JC, Nugent R (2018). Cardiovascular, respiratory, and related disorders: key messages from disease control priorities, 3rd edition. Lancet.

[3] Song PK, Man QQ, Li H, Pang SJ, Jia SS, Li YQ, He L, Zhao WH, Zhang J (2019). Trends in lipids level and dyslipidemia among Chinese adults, 2002-2015. Biomed Environ Sci BES.

[4] Zhang M, Deng Q, Wang L, Huang Z, Zhou M, Li Y, Zhao Z, Zhang Y, Wang L (2018). Corrigendum to "Prevalence of dyslipidemia and achievement of low-density lipoprotein cholesterol targets in Chinese adults: A nationally representative survey of 163,641 adults" [Int. J. Cardiol. 260 (2018) 196–203]. Int J Cardiol.

[5] Opoku S, Gan Y, Fu W, Chen D, Addo-Yobo E, Trofimovitch D, Yue W, Yan F, Wang Z, Lu Z (2019). Prevalence and risk factors for dyslipidemia among adults in rural and urban China: findings from the China National Stroke Screening and prevention project (CNSSPP). BMC Public Health.

[6] Madsen CM, Varbo A, Nordestgaard BG (2017). Extreme high high-density lipoprotein cholesterol is paradoxically associated with high mortality in men and women: two prospective cohort studies. Eur Heart J.

[7] Lu JM, Wu MY, Yang ZM, Zhu Y, Li D, Yu ZB, Shen P, Tang ML, Jin MJ, Lin HB, Shui LM, Chen K, Wang JB (2021). Low LDL-C levels are associated with risk of mortality in a Chinese cohort study. Endocrine.

[8] Alshamiri M, Ghanaim M, Barter P, Chang KC, Li JJ, Matawaran BJ, Santoso A, Shaheen S, Suastika K, Thongtang N (2018). Expert opinion on the applicability of dyslipidemia guidelines in Asia and the Middle East. Int J Gen Med.

[9] Hendrani AD, Adesiyun T, Quispe R, Jones SR, Stone NJ, Blumenthal RS, Martin SS (2016). Dyslipidemia management in primary prevention of cardiovascular disease: current guidelines and strategies. World J Cardiol.

[10] Kuwabara M, Borghi C, Cicero A, Hisatome I, Niwa K, Ohno M, Johnson RJ, Lanaspa MA (2018). Elevated serum uric acid increases risks for developing high LDL cholesterol and hypertriglyceridemia: a five-year cohort study in Japan. Int J Cardiol.

[11] Chen S, Yang H, Chen Y, Wang J, Xu L, Miao M, Xu C (2020). Association between serum uric acid levels and dyslipidemia in Chinese adults: a cross-sectional study and further meta-analysis. Medicine (Baltimore).

[12] Song M, Li N, Yao Y, Wang K, Yang J, Cui Q, Geng B, Chen J, Wang Y, Cheng W, Zhou Y (2019). Longitudinal association between serum uric acid levels and multiterritorial atherosclerosis. J Cell Mol Med.

[13] Cheng F, Yin X, Duan W, Ye R, Zhu Y, Jia C (2019). Different-shaped curves for serum uric acid with and without diabetes: results from China health and retirement longitudinal study. J Diabetes.

[14] Yu TY, Jee JH, Bae JC, Jin SM, Baek JH, Lee MK, Kim JH (2016). Serum uric acid: a strong and independent predictor of metabolic syndrome after adjusting for body composition. Metabolism.

[15] Fang J, Alderman MH (2000). Serum uric acid and cardiovascular mortality the NHANES I epidemiologic follow-up study, 1971-1992. National Health and nutrition examination survey. JAMA.

[16] Peng TC, Wang CC, Kao TW, Chan JY, Yang YH, Chang YW, Chen WL (2015). Relationship between hyperuricemia and lipid profiles in US adults. Biomed Res Int.

[17] Son M, Seo J, Yang S (2020). Association between dyslipidemia and serum uric acid levels in Korean adults: Korea National Health and nutrition examination survey 2016-2017. PLoS One.

[18] Li Y, Tian L, Zheng H, Jia C (2020). Serum uric acid and risk of incident hypercholesterolaemia and hypertriglyceridaemia in middle-aged and older Chinese: a 4-year prospective cohort study. Ann Med.

[19] Ali N, Rahman S, Islam S, Haque T, Molla NH, Sumon AH, Kathak RR, Asaduzzaman M, Islam F, Mohanto NC, Hasnat MA, Nurunnabi SM, Ahmed S (2019). The relationship between serum uric acid and lipid profile in Bangladeshi adults. BMC Cardiovasc Disord.

[20] Lu W, Song K, Wang Y, Zhang Q, Li W, Jiao H, Wang G, Huang G (2012). Relationship between serum uric acid and metabolic syndrome: an analysis by structural equation modeling. J Clin Lipidol.

[21] Keenan T, Blaha MJ, Nasir K, Silverman MG, Tota-Maharaj R, Carvalho JA, Conceição RD, Blumenthal RS, Santos RD (2012). Relation of uric acid to serum levels of high-sensitivity C-reactive protein, triglycerides, and high-density lipoprotein cholesterol and to hepatic steatosis. Am J Cardiol.

[22] Liu W, Liu W, Wang S, Tong H, Yuan J, Zou Z, Liu J, Yang D, Xie Z (2021). Prevalence and risk factors associated with hyperuricemia in the Pearl River Delta, Guangdong Province, China. Risk Manag Healthc Policy.

[23] Li NF, Wang HM, Yang J, Zhou L, Yao XG, Hong J (2009). Serum uric acid is associated with metabolic risk factors for cardiovascular disease in the Uygur population. Appl Physiol Nutr Metab.

[24] Li L, Song Q, Yang X (2019). Lack of associations between elevated serum uric acid and components of metabolic syndrome such as hypertension, dyslipidemia, and T2DM in overweight and obese Chinese adults. J Diabetes Res.

[25] Kuwabara M, Niwa K, Hisatome I, Nakagawa T, Roncal-Jimenez CA, Andres-Hernando A, Bjornstad P, Jensen T, Sato Y, Milagres T, Garcia G, Ohno M, Lanaspa MA, Johnson RJ (2017). Asymptomatic hyperuricemia without comorbidities predicts Cardiometabolic diseases: five-year Japanese cohort study. Hypertension.

[26] Zhao X, Hong F, Yin J, Tang W, Zhang G, Liang X, et al. Cohort profile: the China multi-ethnic cohort (CMEC) study. Int J Epidemiol. 2020; 10.1093/ije/dyaa185 PMC8271196 33232485

[27] Zhang X, Hong F, Qin Z, Liu L, Yang J, Tang X, et al. Resting heart rate is associated with the risk of metabolic syndrome and its components among Dong adults in southwest China: Cross-sectional findings of the China Multi-Ethnic Cohort Study. Diabetes Metab Res Rev. 2021:e3475. 10.1002/dmrr.3475 34036712

[28] Zhao SP (2016). Amendment of the low-density lipoprotein cholesterol target in the 'Chinese guidelines for the prevention and treatment of adult Dyslipidemia': opinion. Chronic Dis Transl Med.

[29] Su P, Hong L, Zhao Y, Sun H, Li L (2015). Relationship between hyperuricemia and cardiovascular disease risk factors in a Chinese population: a cross-sectional study. Med Sci Monit.

[30] Liu L, Yuan Z, Zhang L, Zhang X (2021). Association between serum uric acid levels and the risk of prevalent cardiovascular diseases in ethnic groups, Guizhou. Modern Prev Med.

[31] Wang J, Zhang L, Wang F, Liu L, Wang H (2014). Prevalence, awareness, treatment, and control of hypertension in China: results from a national survey. Am J Hypertens.

[32] Society CD (2021). Guideline for the prevention and treatment of type 2 diabetes mellitus in China (2020 edition). Chin J Diabetes Mellitus.

[33] Babio N, Martínez-González MA, Estruch R, Wärnberg J, Recondo J, Ortega-Calvo M, Serra-Majem L, Corella D, Fitó M, Ros E, Becerra-Tomás N, Basora J, Salas-Salvadó J (2015). Associations between serum uric acid concentrations and metabolic syndrome and its components in the PREDIMED study. Nutr Metab Cardiovasc Dis.

[34] Cardoso AS, Gonzaga NC, Medeiros CC, Carvalho DF (2013). Association of uric acid levels with components of metabolic syndrome and non-alcoholic fatty liver disease in overweight or obese children and adolescents. J Pediatr.

[35] Stone NJ, Robinson JG, Lichtenstein AH, Bairey MC, Blum CB, Eckel RH, Goldberg AC, Gordon D, Levy D, Lloyd-Jones DM (2014). 2013 ACC/AHA guideline on the treatment of blood cholesterol to reduce atherosclerotic cardiovascular risk in adults: a report of the American College of Cardiology/American Heart Association task force on practice guidelines. J Am Coll Cardiol.

[36] Cesaro A, Schiavo A, Moscarella E, Coletta S, Conte M, Gragnano F, Fimiani F, Monda E, Caiazza M, Limongelli G, D’Erasmo L, Riccio C, Arca M, Calabrò P (2021). Lipoprotein(a): a genetic marker for cardiovascular disease and target for emerging therapies. J Cardiovasc Med (Hagerstown).

[37] Gragnano F, Fimiani F, Di Maio M, Cesaro A, Limongelli G, Cattano D, Calabrò P (2019). Impact of lipoprotein(a) levels on recurrent cardiovascular events in patients with premature coronary artery disease. Intern Emerg Med.

[38] Fogacci F, Cicero AF, D'Addato S, D'Agostini L, Rosticci M, Giovannini M, Bertagnin E, Borghi C (2017). Serum lipoprotein(a) level as long-term predictor of cardiovascular mortality in a large sample of subjects in primary cardiovascular prevention: data from the Brisighella heart study. Eur J Intern Med.

[39] Adamopoulos D, Vlassopoulos C, Seitanides B, Contoyiannis P, Vassilopoulos P (1977). The relationship of sex steroids to uric acid levels in plasma and urine. Acta Endocrinol.

[40] Goek ON, Köttgen A, Hoogeveen RC, Ballantyne CM, Coresh J, Astor BC (2012). Association of apolipoprotein A1 and B with kidney function and chronic kidney disease in two multiethnic population samples. Nephrol Dial Transplant.

[41] Zheng R, Ren P, Chen Q, Yang T, Chen C, Mao Y (2017). Serum uric acid levels and risk of incident hypertriglyceridemia: a longitudinal population-based epidemiological study. Ann Clin Lab Sci.

[42] Piepoli MF, Hoes AW, Agewall S, Albus C, Brotons C, Catapano AL, Cooney MT, Corrà U, Cosyns B, Deaton C (2016). 2016 European Guidelines on cardiovascular disease prevention in clinical practice: The Sixth Joint Task Force of the European Society of Cardiology and Other Societies on Cardiovascular Disease Prevention in Clinical Practice (constituted by representatives of 10 societies and by invited experts) Developed with the special contribution of the European Association for Cardiovascular Prevention & Rehabilitation (EACPR). Eur Heart J.

[43] Celis-Morales CA, Welsh P, Lyall DM, Steell L, Petermann F, Anderson J, Iliodromiti S, Sillars A, Graham N, Mackay DF (2018). Associations of grip strength with cardiovascular, respiratory, and cancer outcomes and all cause mortality: prospective cohort study of half a million UK biobank participants. BMJ.

[44] Gazzola K, Snijder MB, Hovingh GK, Stroes E, Peters R, van den Born BH (2018). Ethnic differences in plasma lipid levels in a large multiethnic cohort: the HELIUS study. J Clin Lipidol.

[45] Xi Y, Niu L, Cao N, Bao H, Xu X, Zhu H, Yan T, Zhang N, Qiao L, Han K, Hang G, Wang W, Zhang X (2020). Prevalence of dyslipidemia and associated risk factors among adults aged ≥35 years in northern China: a cross-sectional study. BMC Public Health.

[46] Micek A, Grosso G, Polak M, Kozakiewicz K, Tykarski A, Puch WA, Drygas W, Kwaśniewska M, Pająk A (2018). Association between tea and coffee consumption and prevalence of metabolic syndrome in Poland - results from the WOBASZ II study (2013-2014). Int J Food Sci Nutr.

[47] Pan L, Yang Z, Wu Y, Yin RX, Liao Y, Wang J, Gao B, Zhang L (2016). The prevalence, awareness, treatment and control of dyslipidemia among adults in China. Atherosclerosis.

[48] Desquilbet L, Mariotti F (2010). Dose-response analyses using restricted cubic spline functions in public health research. Stat Med.

[49] Rahimi-Sakak F, Maroofi M, Rahmani J, Bellissimo N, Hekmatdoost A (2019). Serum uric acid and risk of cardiovascular mortality: a systematic review and dose-response meta-analysis of cohort studies of over a million participants. BMC Cardiovasc Disord.

[50] Hu L, Hu G, Xu BP, Zhu L, Zhou W, Wang T, et al. U-Shaped Association of Serum Uric Acid With All-Cause and Cause-Specific Mortality in US Adults: A Cohort Study. J Clin Endocrinol Metab. 2020;105(3):–e609. 10.1210/clinem/dgz068. 10.1210/clinem/dgz068 31650159

[51] Tseng WC, Chen YT, Ou SM, Shih CJ, Tarng DC. U-Shaped Association Between Serum Uric Acid Levels With Cardiovascular and All-Cause Mortality in the Elderly: The Role of Malnourishment. J Am Heart Assoc. 2018;7(4) 10.1161/JAHA.117.007523. 10.1161/JAHA.117.007523 PMC5850189 29440009

[52] Lisa Y (2015). Analysis of the hyperuricemia and its related factors in a part of Buyi, Miao,Han adults from the south of Guizhou province. Guizhou Medical University.

[53] Aihemaitijiang S, Zhang Y, Zhang L, Yang J, Ye C, Halimulati M, et al. The Association between Purine-Rich Food Intake and Hyperuricemia: A Cross-Sectional Study in Chinese Adult Residents. Nutrients. 2020;12(12) 10.3390/nu12123835. 10.3390/nu12123835 PMC7765492 33334038

[54] Vassalle C, Mazzone A, Sabatino L, Carpeggiani C (2016). Uric Acid for Cardiovascular Risk: Dr. Jekyll or Mr. Hide?. Diseases.

[55] Mann S, Beedie C, Jimenez A (2014). Differential effects of aerobic exercise, resistance training and combined exercise modalities on cholesterol and the lipid profile: review, synthesis and recommendations. Sports Med.

[56] Hou Y, Ma R, Gao S, Kaudimba KK, Yan H, Liu T, Wang R (2021). The effect of low and moderate exercise on hyperuricemia: protocol for a randomized controlled study. Front Endocrinol (Lausanne).

